# NUTRITIONAL ASSESSMENT AND ITS IMPACT ON QUALITY OF LIFE IN PATIENTS WITH INFLAMMATORY BOWEL DISEASE IN COLOMBIA: NUTRIBD - COL

**DOI:** 10.1590/S0004-2803.24612025-090

**Published:** 2026-01-09

**Authors:** Viviana PARRA-IZQUIERDO, Carlos Augusto CUADROS, Ginary ORDUZ-DIAZ, Paula Daniela BONILLA-RIBERO, Oscar Mariano PINTO, Cristian Fabian FLOREZ, Juan Javier ACEVEDO, Julian FERREIRA, Vanessa DURAN, Luis Felipe MURCIA, Juan Sebastian FRIAS-ORDOÑEZ

**Affiliations:** 1 International Hospital of Colombia, Center of Excellence in Inflammatory Bowel Disease, Bucaramanga, Colombia.; 2 El Bosque University, INMUBO Cellular and Molecular Immunology Research Group, Bogotá, Colombia.; 3 Cardiovascular Foundation of Colombia, Medical and Surgical Specialties Research Group, Floridablanca, Colombia.

**Keywords:** Inflammatory bowel diseases, nutritional status, quality of life, malnutrition, enteral nutrition, nutritional assessment, Doenças inflamatórias intestinais, estado nutricional, qualidade de vida, desnutrição, nutrição enteral, avaliação nutricional

## Abstract

**Background:**

Inflammatory bowel disease (IBD) significantly affects patients’ nutritional status and quality of life (QoL). Nutritional deficiencies are frequent and may worsen disease outcomes. The aim of this study is to assess the association between nutritional status and quality of life in patients with IBD.

**Methods:**

A cross-sectional study was conducted in a specialised IBD centre. Nutritional status was evaluated using the MUST and MIRT tools in adults and the “Strong Kids” scale in paediatrics. An individualised nutritional support protocol was implemented. Quality of life was assessed with the EQ-5D and IBDQ-32 instruments.

**Results:**

Among 90 patients, 26.6% required hospitalisation, predominantly adults and females. The mean age was 36.7 years. Crohn’s disease (CD) was more prevalent (58.3%), and 83.3% received biological therapy. Malnutrition risk in hospitalised patients with ulcerative colitis (UC) and CD was significantly increased, with odds ratios of 8.33 (95%CI: 1.16-27) and 25 (95%CI: 11.6-45.2), respectively. All hospitalised patients with severe disease presented with malnutrition. In the paediatric group, 87.5% received enteral nutrition and 25% required parenteral nutrition. Nutritional support improved nutritional status in 75% of cases within three months. Malnutrition was significantly associated with poorer QoL, particularly affecting mobility, self-care, and daily activities (*P*<0.05).

**Conclusion:**

Malnutrition has a substantial negative impact on quality of life in IBD patients. Early detection using tools such as MUST, MIRT, and strong kids is essential. Integrating systematic nutritional screening and tailored support into routine clinical practice is imperative to improve outcomes and promote recovery.

## HIGHLIGHTS

•Descriptive, cross-sectional study in a Colombian IBD Centre of Excellence (adults and paediatrics, inpatients and outpatients).

•To assess the association between nutritional status and quality of life in patients with inflammatory bowel disease (IBD).

•MUST and MIRT (adults); STRONG Kids (paediatrics); EQ-5D and IBDQ-32 for quality of life.

•Malnutrition was present in >60% of hospitalised patients; strongly associated with poor quality of life. Nutritional support improved outcomes.

•Study conducted by a team of gastroenterologists, nutritionists, paediatricians, and clinical researchers.

•First study in Colombia evaluating nutritional status and QoL in IBD patients using structured screening and follow-up.

•Recommends routine nutritional screening and support as essential components of IBD management.

## RESUMO

**Contexto:**

As doenças inflamatórias intestinais (DII) afetam significativamente o estado nutricional e a qualidade de vida (QV) dos pacientes. As deficiências nutricionais são frequentes e podem agravar os desfechos clínicos da doença. O objetivo deste estudo foi avaliar a associação entre o estado nutricional e a qualidade de vida em pacientes com DII.

**Métodos:**

Estudo transversal realizado em um centro especializado em DII. O estado nutricional foi avaliado utilizando os instrumentos MUST e MIRT em adultos e a escala “Strong Kids” em pacientes pediátricos. Foi implementado um protocolo individualizado de suporte nutricional. A qualidade de vida foi avaliada com os instrumentos EQ-5D e IBDQ-32.

**Resultados:**

Entre 90 pacientes, 26,6% necessitaram de hospitalização, predominantemente adultos e do sexo feminino. A média de idade foi de 36,7 anos. A doença de Crohn (DC) foi mais prevalente (58,3%), e 83,3% dos pacientes recebiam terapia biológica. O risco de desnutrição em pacientes hospitalizados com retocolite ulcerativa (RCU) e DC foi significativamente aumentado, com razões de chances de 8,33 (IC95%: 1,16-27) e 25 (IC95%: 11,6-45,2), respectivamente. Todos os pacientes hospitalizados com doença grave apresentaram desnutrição. No grupo pediátrico, 87,5% receberam nutrição enteral e 25% necessitaram de nutrição parenteral. O suporte nutricional melhorou o estado nutricional em 75% dos casos em três meses. A desnutrição foi significativamente associada a pior qualidade de vida, afetando especialmente a mobilidade, o autocuidado e as atividades diárias (*P*<0,05).

**Conclusão:**

A desnutrição tem um impacto negativo importante na qualidade de vida dos pacientes com DII. A detecção precoce por meio de ferramentas como MUST, MIRT e Strong Kids é essencial. A integração de triagem nutricional sistemática e suporte individualizado à prática clínica de rotina é fundamental para melhorar os desfechos e promover a recuperação.

## INTRODUCTION

Inflammatory bowel disease (IBD), encompassing Crohn’s disease (CD) and ulcerative colitis (UC), represents a growing global health challenge, with rising incidence in newly industrialised countries^1^. Beyond chronic intestinal inflammation, malnutrition is one of its most critical and underrecognised complications, significantly impacting clinical outcomes and patients’ quality of life^2^.

Malnutrition in IBD arises from a multifactorial interplay, including impaired nutrient absorption, reduced dietary intake, and adverse effects of pharmacological therapies^3^ ^,^ ^4^. Up to 75% of patients in active disease phases are malnourished, and even during remission, prevalence can reach 38%^5^ ^,^ ^6^. Sarcopenia-marked by loss of muscle mass and strength-has also emerged as a frequent complication, worsening disease progression and diminishing therapeutic response^7^ ^,^ ^8^. Reported malnutrition prevalence in IBD varies widely, ranging from 5.7% to 82.8%, depending on the population and assessment methods used^6^. Despite significant advances in IBD management, malnutrition remains a persistent and underestimated barrier to optimal care, even in settings with high access to specialised services^5^. Its consequences include increased hospitalisation rates, postoperative complications, and impaired quality of life^9^. Malnutrition in patients with IBD imposes a substantial clinical, economic, and social burden. Hospitalised IBD patients are 2.9-3.1 times more likely to present with protein-calorie malnutrition, which is linked to longer hospital stays, higher readmission and mortality rates, and increased healthcare costs. Although enteral and parenteral nutritional support may reduce readmissions, it has also been associated with higher mortality and hospital expenditures, underscoring the need for optimised interventions^10^.

To counter these effects, systematic nutritional assessment is essential. Tools such as the malnutrition universal screening tool (MUST) and the malnutrition inflammation risk tool (MIRT) for adults, alongside “strong kids” for paediatric populations, have proven effective in early risk detection among IBD patients^9^. Malnutrition in patients with IBD has been associated with impaired immune response, increased risk of postoperative complications, and reduced efficacy of pharmacological therapies, including biologics-highlighting its role as a key modifiable factor in disease prognosis^11^ ^-^ ^13^. Although regional data on IBD-related malnutrition remain scarce, it is widely recognised as a frequent complication driven by chronic inflammation, malabsorption, and treatment side effects-warranting early detection and intervention. Also, no studies in Colombia have comprehensively assessed the nutritional status of IBD patients or its relationship with quality of life. This study seeks to address this knowledge gap by evaluating the nutritional status and self-perceived quality of life in patients treated at the Centre of Excellence for Inflammatory Bowel Disease at the Hospital Internacional de Colombia (HIC), with a particular focus on adults at risk of malnutrition. We hypothesise that an increased risk of malnutrition is independently associated with lower quality of life, irrespective of IBD subtype or care setting (inpatient vs. outpatient). This research was designed and executed as a multidisciplinary study, integrating the expertise of gastroenterologists, nutritionists, paediatric specialists, epidemiologists, and clinical researchers. Each discipline contributed to the conceptualisation, data collection, nutritional assessment, quality of life evaluation, and statistical analysis. This collaborative framework was essential to comprehensively address the complex interaction between nutritional status and quality of life in both adult and paediatric populations with IBD.

## METHODS

A descriptive, cross-sectional observational study was conducted at the Inflammatory Bowel Disease Centre of Excellence at the Hospital Internacional de Colombia (HIC) between January and December 2023. Consecutive paediatric and adult patients with a confirmed diagnosis of inflammatory bowel disease (IBD), including Crohn’s disease (CD) and ulcerative colitis (UC), were prospectively enrolled during routine inpatient or outpatient visits. A total of 102 patients were initially screened; 12 were excluded due to incomplete nutritional or clinical data or refusal to participate, leaving 90 patients for final analysis. Of these, 78 completed the 3-month nutritional follow-up, while 12 were lost to follow-up due to relocation or discontinuation of care. Patients were excluded if they declined to provide informed consent, had incomplete clinical or nutritional data, or were lost to follow-up before completing the nutritional reassessment.

### Nutritional intervention protocol

All patients received structured and individualised nutritional care, implemented by dietitians in collaboration with gastroenterologists. Interventions followed a step-wise algorithm:

1.Dietary counselling with tailored macronutrient and micronutrient recommendations.

2.Oral nutritional supplementation when caloric intake was <75% of estimated requirements.

3.Enteral nutrition (via nasogastric or nasojejunal tube, or orally with hypercaloric, high-protein polymeric formulas) when oral intake remained insufficient.

4.Parenteral nutrition reserved for patients with severe malabsorption, intestinal failure, or contraindication to enteral feeding.

5.Monitoring and follow-up: anthropometry, biochemical parameters, and functional assessments at baseline and at 3 months.

### Assessment tools, data collection and statistical analysis

A total of 90 patients were evaluated. The nutritional screening was tailored by age group: adults were assessed using the Malnutrition Universal Screening Tool (MUST)^14^ and the Malnutrition Inflammation Risk Tool (MIRT)^15^, whereas paediatric patients were evaluated with the Strong Kids screening tool^16^. Additionally, anthropometric standards from the World Health Organization (WHO) were applied for children under two years of age, and those from the Centers for Disease Control and Prevention (CDC) for children older than two years.

In adults, the MUST^14^ evaluates body mass index (BMI), unintentional weight loss over the previous six months, and the presence of acute illness to classify patients into low (0 points), moderate (1 point), or high (≥2 points) malnutrition risk. The MIRT^15^ incorporates BMI, recent weight loss, and serum C-reactive protein (CRP) levels to integrate both nutritional and inflammatory components of risk. Parameters related to sarcopenia were also assessed, including muscle strength, muscle mass, and physical performance, according to international consensus definitions.

The strong kids tool^16^, used in paediatric patients, assigns a risk score based on four clinical criteria: high-risk underlying disease (2 points), reduced nutritional intake in recent days (1 point), recent weight loss or underweight (1 point), and impaired growth (1 point). Scores range from 0 to 5, categorising nutritional risk as low (0), moderate (1-3), or high (4-5).

Quality of life (QoL) in adult patients was assessed using the Inflammatory Bowel Disease Questionnaire (IBDQ-32)^17^, which consists of 32 items distributed across four domains: intestinal symptoms (10 items), systemic symptoms (5 items), emotional well-being (12 items), and social functioning (5 items). Each item is rated on a 7-point Likert scale, with 1 representing the worst and 7 the best quality of life, yielding a total score between 32 and 224. Additionally, the EQ-5D questionnaire^18^ was used due to its validated utility in capturing multidimensional QoL outcomes across chronic disease populations and enabling comparisons between health states.

The nutritional screening tools used in this study-MUST^14^, MIRT^15^, and strong kids^16^-have been previously translated and adapted for use in Spanish-speaking populations, with documented applicability in Latin American clinical settings^19^ ^,^ ^20^.

Data were collected prospectively at baseline and after a 3-month follow-up for patients receiving nutritional intervention. Nutritional interventions were individualised and implemented by a multidisciplinary team, including registered dietitians, gastroenterologists, and nursing staff. To minimise selection bias, consecutive sampling was employed, and patients with incomplete records or who declined participation were excluded. Information bias was reduced through prospective data collection by trained clinical staff using standardised forms. Disease severity at hospital admission was defined using validated clinical indices: the Mayo Score for ulcerative colitis, the Harvey-Bradshaw Index for Crohn’s disease, and, in children, PUCAI or PCDAI as appropriate. Complementary laboratory parameters such as serum albumin, C-reactive protein (CRP), and hemoglobin levels were also considered to corroborate clinical assessments.

Statistical analyses were performed using SPSS version 25.0. Descriptive statistics summarised demographic and clinical characteristics, including age, sex, disease duration, and IBD activity scores. A multivariate logistic regression model was constructed to identify independent predictors of malnutrition, adjusting for age, sex, type of IBD (CD vs UC), hospitalisation status, disease activity, and use of biologics. Odds ratios (OR) and 95% confidence intervals (CI) were calculated. Associations between nutritional status and QoL outcomes (EQ-5D and IBDQ-32) were analysed using chi-square tests for categorical variables and either Student’s t-test or the Mann-Whitney U test for continuous variables, depending on distribution. A *P*-value <0.05 was considered statistically significant. 

This study was conducted in accordance with the ethical principles set forth in the declaration of helsinki by the world medical association. Informed consent was obtained from all participants or legal guardians. The study protocol was reviewed and approved by the Clinical Research Ethics Committee of the Hospital Internacional de Colombia, ensuring adherence to national and international standards of biomedical research and patient confidentiality.

### Ethical considerations

The authors affirm that this study was conducted in accordance with the ethical standards of the World Medical Association (Declaration of Helsinki) for research involving human subjects. Informed consent was obtained from all participants, and all privacy rights were respected. The study was approved by the Clinical Research Ethics Committee of the Hospital Internacional de Colombia.

## RESULTS

### Study population

Over a 12-month period, 102 patients were screened, of whom 90 met the eligibility criteria and were included in the analysis. A total of 78 patients (86.7%) completed the three-month nutritional follow-up, while 12 (13.3%) were excluded from the longitudinal analysis due to incomplete data or loss to follow-up. Overall, 26.6% of the cohort required hospitalisation for disease exacerbation, predominantly adults and females (Table 1). The mean age of hospitalised patients was 36.7 years (range:4-81 years; SD:32.6), underscoring the wide age spectrum affected by disease severity. Notably, 58.3% of the cohort had a diagnosis of Crohn’s disease (CD), and a striking 83.3% of hospitalised individuals initiated biologic therapy during the study period (Table 1 and Table 2), reflecting both the burden of severe disease and the high therapeutic demands in this population.

**TABLE 1:** Clinical, nutritional and therapeutic characteristics of hospitalized adult patients with inflammatory bowel disease.

Variable	Ulcerative colitis (n=9)	Crohn’s disease (n=7)	***P*-value**
**Female, n(%)**	2 (22.2)	4 (57.2)	0.300
**Age, mean (range; SD)**	46 (31.5-60.5; 18.8)	52.7 (31-81; 17.6)	0.542
**BMI, mean (range; SD)**	22.8 (15.7-27.8; 3.95)	20.7 (15-26; 3.29)	0.285
**Therapy, n(%)**			
Mesalazine	9 (100)	1 (14.3)	<0.001
Corticosteroids	1 (11.1)	1 (14.3)	1.000
Biologics	7 (77.7)	4 (57.2)	0.586
JAK inhibitors	0 (0)	2 (28.5)	0.171
**Disease characteristics, n(%)**			
Prior IBD surgery	0 (0)	3 (42.8)	0.057
IBD-related hospitalization	0 (0)	5 (71.4)	0.010
Extraintestinal manifestations	0 (0)	0 (0)	-
**Disease activity**			
Mayo score - moderate	4 (44.4)	0	0.090
Mayo score - severe	5 (55.5)	0	0.029
Harvey-bradshaw - moderate	0	6 (85.7)	<0.001
Harvey-bradshaw - severe	0	1 (14.3)	0.429
**Nutritional diagnosis, n(%)**			
Severe malnutrition	0	0	-
Moderate malnutrition	0	0	-
Mild malnutrition	2 (22.2)	1 (14.3)	1.000
Eutrophic	4 (44.4)	5 (71.4)	0.586
Healthy weight	0	1 (14.3)	0.429
Overweight	2 (22.2)	0	0.476
Obesity	0	0	-
**Nutritional support, n(%)**			
Nursing advice	9 (100)	7 (100)	-
Nutritionist counseling	2 (22.2)	1 (14.2)	1.000
Partial enteral nutrition	1 (11.1)	0	1.000
Total enteral nutrition	0	0	-
Parenteral nutrition	0	0	-

Notes: *P*-values were calculated using Fisher’s exact test or Mann-Whitney U test, as appropriate. Statistical significance was set at *P*<0.05. Missing *P*-values indicate variables with no variance between groups. IBD: inflammatory bowel disease. BMI: body mass index. SD: standard deviation. CU: ulcerative colitis. CD: crohn’s disease JAK: janus kinase.

**TABLE 2:** Clinical, nutritional and therapeutic characteristics of hospitalized pediatric patients with inflammatory bowel disease.

Variable	Ulcerative colitis (n=1)	Crohn’s disease (n=7)	***P*-value**
**Female, n (%)**	1 (100)	5 (71.4)	1.000
**Age, mean (range; SD)**	17.9	11.29 (4-20; 4.95)	-
**BMI, mean (range; SD)**	20.0	16.3 (13.6-18.9; 2.86)	-
**Therapy, n(%)**			
Mesalazine	0	0	-
Corticosteroids	0	0	-
Biologics	0	7 (100)	0.048
JAK inhibitors	1 (100)	0	0.048
**Disease characteristics, n(%)**			
Prior IBD surgery	0	3 (42.8)	0.429
IBD-related hospitalization	1 (100)	7 (100)	-
Extraintestinal manifestations	0	0	-
**Disease activity**			
PUCAI - moderate	1 (100)	0	0.048
PUCAI - severe	0	0	-
PCDAI - moderate	0	3 (42.8)	0.429
PCDAI - severe	0	4 (57.2)	0.190
**Nutritional diagnosis, n(%)**			
Severe malnutrition	0	1 (14.3)	1.000
Moderate malnutrition	0	2 (28.6)	1.000
Mild malnutrition	0	1 (14.3)	1.000
Eutrophic	0	1 (14.3)	1.000
Healthy weight	1 (100)	1 (14.3)	0.048
Overweight	0	0	-
Obesity	0	0	-
**Nutritional support, n(%)**			
Nursing advice	1 (100)	7 (100)	-
Nutritionist counseling	1 (100)	7 (100)	-
Partial enteral nutrition	0	2 (28.6)	1.000
Total enteral nutrition	1 (100)	2 (28.6)	0.190
Parenteral nutrition	0	3 (42.9)	0.429

Notes: *P*-values were calculated using Fisher’s exact test. Statistical analysis was limited by small sample size and asymmetry. Variables with no variance across groups are marked with “-”. Statistical significance was defined as *P*<0.05 IBD: inflammatory bowel disease. BMI: body mass index. SD: standard deviation. PUCAI: pediatric ulcerative colitis activity index. PCDAI: pediatric crohn’s disease activity index. JAK: janus kinase.

### Nutritional assessment - adult population

**FIGURE 1:**
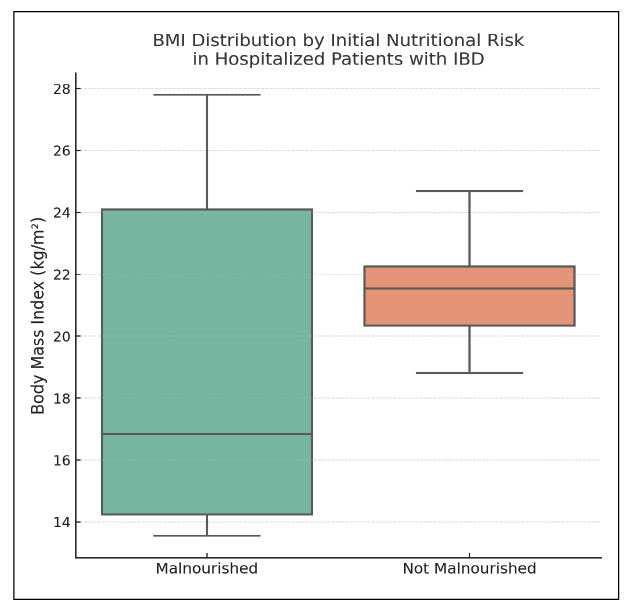
Body mass index by initial nutritional risk in hospitalized patients with inflammatory bowel disease.

Hospitalised patients with ulcerative colitis (UC) demonstrated a significantly elevated risk of malnutrition, with an odds ratio (OR) of 8.33 (95%CI: 1.16-27). This risk was even more pronounced in those with Crohn’s disease (CD), where the OR reached 25 (95%CI: 11.6-45.2), as shown in Figure 1. All hospitalised adults exhibited biochemical abnormalities, most notably moderate-to-severe hypoalbuminaemia, and 25% were diagnosed with sarcopenia. Nutritional support was provided to 49% of the total adult cohort, and importantly, 100% of hospitalised patients with severe disease were found to be malnourished. Following targeted nutritional intervention, more than 80% of these patients experienced substantial improvement in their nutritional status.

Boxplot comparing body mass index (BMI) in hospitalized patients with inflammatory bowel disease (IBD), stratified by nutritional risk status at admission. Although median BMI was lower in those classified as malnourished (Group 1), the difference was not statistically significant (*P*=0.12, Mann-Whitney U test). The data highlight a clinically relevant trend toward lower nutritional reserves among high-risk individuals, warranting routine screening and intervention. Notes: the whiskers represent the minimum and maximum values within 1.5 times the interquartile range. No extreme outliers were detected. BMI was assessed at the time of admission, prior to any nutritional support. BMI: body mass index. IBD: inflammatory bowel disease.

In the outpatient adult cohort (Table 3), 20 individuals underwent formal nutritional screening. Of these, 30% were identified as being at high risk of malnutrition using the Malnutrition Universal Screening Tool (MUST), and 25% had a clinically significant nutritional risk as defined by a MIRT score ≥3. These findings underscore the hidden yet critical burden of nutritional impairment in patients who are not acutely hospitalised, highlighting the importance of routine screening even in the ambulatory setting.

**TABLE 3:** Prevalence of nutritional risk in ambulatory adults with inflammatory bowel disease (n=20).

Screening tool	Patients at risk, n(%)	95% Confidence interval
MUST (≥1)	9/28 (32.1%)	17.9% - 50.7%
MIRT (≥3)	6/28 (21.4%)	10.2% - 39.5%

Notes: the malnutrition universal screening tool (MUST) identified one-third of the outpatient cohort as being at high nutritional risk. A MIRT score ≥3, indicating clinically significant nutritional compromise, was found in over one-fifth of patients. These findings emphasize the importance of systematic nutritional screening in non-hospitalized IBD populations. MUST: malnutrition universal screening tool. MIRT: multidimensional inflammatory-related tool.

**TABLE 4:** Association between nutritional status and quality of life in patients with inflammatory bowel disease (multivariate logistic regression).

Quality of life outcome (IBDQ-32 / EQ-5D)	Adjusted OR for poor qol in malnourished patients (95%CI)	***P*-value**
IBDQ-32 total score<median	2.74 (1.23 - 6.11)	0.014
Intestinal symptoms (IBDQ-32 domain)	2.15 (1.08 - 4.28)	0.028
Emotional well-being (IBDQ-32 domain)	1.97 (1.01 - 3.84)	0.042
Social functioning (IBDQ-32 domain)	2.65 (1.21 - 5.81)	0.014
EQ-5D global score < population median	2.88 (1.35 - 6.16)	0.005

Notes: multivariate logistic regression adjusted for age, sex, type of IBD (Crohn’s disease vs. ulcerative colitis), hospitalisation status, and use of biological therapy. QoL: quality of life. IBDQ-32: inflammatory bowel disease questionnaire (32-item version). EQ-5D: EuroQol 5-Dimensions. OR >1 indicates increased odds of impaired QoL among malnourished patients.

### Paediatric population

Among hospitalised paediatric patients, 100% received nutritional support as part of their clinical management. Of these, 62.5% exhibited some degree of malnutrition, while 37.5% were categorised as having high nutritional risk. Within this cohort, 25% required parenteral nutrition, and 87.5% received enteral nutrition, predominantly using hypercaloric and high-protein formulas. Notably, during hospitalisation, 87.5% of patients demonstrated weight gain and clinical improvement in nutritional status, with 75% achieving a normal nutritional profile at the 3-month follow-up.

However, one critically ill paediatric patient with severe chronic malnutrition and refractory disease, unresponsive to multiple lines of medical therapy, died in the early postoperative period following a colectomy. This tragic outcome underscores the life-threatening nature of nutritional failure in complex paediatric IBD and highlights the urgent need for early, aggressive, and individualised nutritional strategies.

### Quality of life assessment - IBDQ-32

**FIGURE 2:**
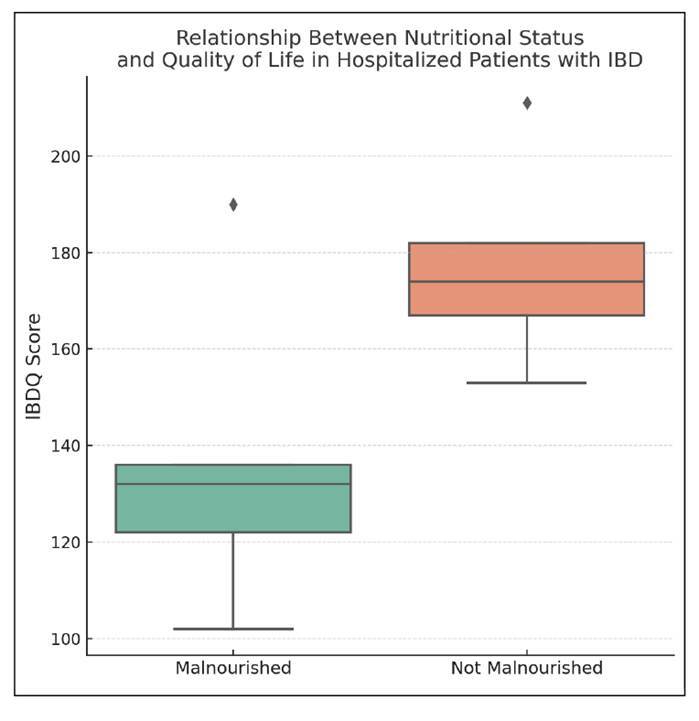
Relationship between nutritional status and quality of life in hospitalized patients with inflammatory bowel disease.

Malnourished patients consistently exhibited lower quality of life scores compared with well-nourished counterparts. After adjusting for age, sex, IBD subtype, hospitalisation, and use of biologics, malnutrition remained an independent predictor of impaired QoL, with significantly higher odds of reduced IBDQ-32 total scores and deficits in intestinal symptoms, emotional well-being, and social functioning (Table 4). Complementary analyses further showed severe impairments in mobility (*P*=0.278; 95%CI: 2.03-64.1), self-care (*P*=0.003; 95%CI: 9.45-90.5), and daily activities (*P*=0.146; 95%CI: 1.14-58.2). Worse nutritional profiles were also linked to heightened pain perception (*P*=0.197; 95%CI: 0.53-53.3) and significantly lower visual analogue scale scores (*P*=0.012; 95%CI: 22.9-88.4) (Figure 2). Taken together, these results confirm the robust and independent association between nutritional status and health-related quality of life in IBD, highlighting early nutritional intervention as a central pillar of comprehensive disease management.

Boxplot comparing disease-specific quality of life, measured by the Inflammatory Bowel Disease Questionnaire (IBDQ), between hospitalized patients with and without nutritional risk at admission. Patients classified as malnourished showed a lower median IBDQ score compared to their well-nourished counterparts. Note: Although the trend is clinically relevant, the difference did not reach statistical significance (*P*=0.095, Mann-Whitney U test). IBDQ: inflammatory bowel disease questionnaire. IBD: inflammatory bowel disease.

## DISCUSSION

This study highlights the strong association between malnutrition and impaired quality of life in IBD, independent of disease type or care setting. Importantly, after adjusting for age, sex, hospitalisation, and biologic use, malnutrition remained a significant predictor of lower QoL scores. This finding underscores malnutrition as an independent determinant of outcomes rather than merely a correlate of disease severity.

Mechanistically, the bidirectional relationship between inflammation and malnutrition in IBD is mediated by proinflammatory cytokines (e.g., TNF, IL-6, IL-17), gut microbiota dysbiosis, and protein depletion^21^. Chronic inflammation impairs nutrient absorption, increases intestinal permeability, and promotes muscle catabolism, while dysbiosis exacerbates immune dysregulation and mucosal damage-contributing to a vicious cycle that perpetuates both malnutrition and disease activity^22^ ^,^ ^23^.

Among ambulatory adult patients, 30% were identified as being at high nutritional risk using the MUST tool, and 25% had a clinically relevant score on the MIRT (≥3 points), supporting the necessity of systematic screening even outside hospital settings. The MUST and MIRT tools have emerged as reliable instruments in assessing nutritional risk in IBD. MUST, which evaluates body mass index, unintentional weight loss, and the impact of acute illness, has shown moderate to high sensitivity in identifying malnutrition compared with GLIM criteria^5^ ^,^ ^24^. Meanwhile, MIRT adds value by incorporating inflammatory biomarkers such as C-reactive protein (CRP), with a sensitivity of 84% in detecting malnutrition and a demonstrated association with adverse clinical outcomes, including hospitalisation and need for surgery^9^.

These findings reaffirm that both tools are practical and clinically useful, with MUST being widely validated and MIRT offering added sensitivity through the inclusion of systemic inflammation. The early identification of malnutrition using these tools enables timely intervention, potentially mitigating complications and improving disease trajectory.

Our malnutrition prevalence rates are consistent with international cohorts. Studies from Europe and North America report malnutrition rates in IBD patients ranging from 6.1% to 69.7%, depending on disease activity, clinical setting, and diagnostic criteria^25^. A multicentre European study found a disease-related malnutrition (DRM) prevalence of 23%, while 36% of newly diagnosed IBD patients met the ESPEN malnutrition definition^5^. Even in clinical remission, up to 39.5% of patients may be at risk^26^. These findings highlight the global relevance of routine nutritional screening and confirm the applicability of our data in broader contexts.

In the inpatient setting, malnutrition was strongly associated with disease severity. Patients with UC had an odds ratio of 8.33 for malnutrition, while those with CD had an even higher risk (OR=25), reflecting the profound nutritional vulnerability among hospitalised individuals. Furthermore, 100% of patients with severe disease were malnourished, and 25% exhibited sarcopenia. Importantly, nutritional intervention led to clinical improvement in over 80% of cases, underscoring the therapeutic impact of targeted nutritional strategies.

Similarly, hospitalised paediatric patients exhibited alarming rates of malnutrition (62.5%) and high nutritional risk (37.5%), requiring intensive support. The majority received hypercaloric enteral nutrition, with a high response rate in terms of weight gain and improved nutritional status, and 75% achieving normalisation within three months. One fatal case, despite advanced nutritional and medical interventions, illustrates the high-risk nature of severe chronic malnutrition in refractory IBD.

Low body mass index (BMI <22kg/m²) and active disease have been previously identified as independent predictors of malnutrition in adult IBD^27^, findings corroborated by our cohort. These underscore the need for disease-specific screening tools that go beyond conventional criteria used in general populations. The heterogeneity of malnutrition between IBD subtypes also highlights the importance of personalised nutritional strategies^28^.

Our results are consistent with prior literature showing higher malnutrition rates among paediatric patients, attributable to more extensive disease presentations, increased inflammatory burden, malabsorption, and growth failure^29^. The documented delay in pubertal development, higher prevalence of stricturing and fistulising disease in CD, and fulminant colitis in UC add complexity to nutritional management in children.

Critically, this study highlights the profound impact of nutritional status on quality of life. Malnourished patients showed significantly lower scores across all domains of the IBDQ-32, including mobility, self-care, daily activities, and pain perception. These findings reinforce the notion that nutritional care should not be a secondary consideration but an integral component of comprehensive IBD management.

Early implementation of structured nutritional screening and support protocols in IBD can reduce the burden of complications, improve treatment response, and enhance the patient experience. These interventions should be standard practice in both outpatient and hospital-based care. In the Latin American context, evidence on nutrition and quality of life in IBD remains limited. A multicentre Brazilian study found that moderate-to-severe disease activity was associated with impaired quality of life, increased surgical rates, and decreased work productivity in patients with CD and UC^30^. Additionally, research from Mexico demonstrated distinct patterns of gut microbiota composition in IBD patients, highlighting the role of dietary and geographic factors in disease pathogenesis^31^. Emerging evidence suggests that native Latin American food byproducts with anti-inflammatory and prebiotic properties, such as passion fruit, pineapple, and pumpkin, may offer novel therapeutic potential in IBD dietary management^32^. These findings open new avenues for exploring culturally relevant nutritional interventions in the region. The structured nutritional protocol implemented-progressing from counselling to oral, enteral, and parenteral support-proved effective, with three-quarters of patients showing improved nutritional status at 3 months. These results demonstrate the feasibility and clinical impact of systematic, algorithm-based nutritional intervention in IBD care.

A key limitation of this study is the relatively small sample size, particularly within the paediatric subgroup, which may limit the statistical power and generalisability of the findings. While the screening tools employed (MUST, MIRT, Strong Kids)^14^ ^-^ ^16^ are widely used internationally, they have not yet been formally validated in Colombian or broader Latin American populations, which may affect cultural and contextual applicability. Another important limitation is the cross-sectional design, which does not allow for assessment of the long-term impact of nutritional status on disease remission, relapse rates, or sustained quality of life improvements. These results should be interpreted in light of certain limitations, including the limited sample size in the paediatric cohort, the lack of formal validation of screening tools in the local context, and the absence of longitudinal follow-up to evaluate the impact of nutritional interventions on long-term disease outcomes.

Despite promising results, this study also identifies critical areas for further research. firstly, the lack of validated, disease-specific nutritional assessment tools in Spanish remains a major barrier. While tools like the IMPACT-III questionnaire have been adapted for Spanish-speaking paediatric populations^33^, instruments such as the GLIM criteria and Subjective Global Assessment (SGA) require further validation in IBD cohorts. Additionally, structured nutritional interventions, including web-based and multidisciplinary models, have shown promise in improving food-related quality of life^34^, yet remain underexplored in Latin America. Longitudinal studies with larger, multicentre cohorts are urgently needed to validate our findings and assess causality. These should incorporate standardised nutritional protocols and culturally adapted QoL instruments to better capture the full impact of malnutrition in IBD.

## CONCLUSION

Malnutrition is highly prevalent in IBD and exerts a profound, independent effect on quality of life. Early, structured, and multidisciplinary nutritional intervention must be incorporated as a standard of care in both inpatient and outpatient settings. Early screening and timely, individualised nutritional intervention should become standard practice across all stages of disease. Future research must prioritise the validation of disease-specific tools and structured interventions in Spanish-speaking populations to ensure equitable, high-quality care. Failure to systematically address malnutrition may undermine otherwise effective therapeutic strategies. Nutrition must be elevated to a central pillar in IBD management.

## Data Availability

Not applicable - The study did not use research data

## References

[1] Ng SC, Shi HY, Hamidi N, Underwood FE, Tang W, Benchimol EI (2017). Worldwide incidence and prevalence of inflammatory bowel disease in the 21st century: a systematic review of population-based studie. Lancet.

[2] Lichtenstein GR, Loftus E V., Isaacs KL, Regueiro MD, Gerson LB, Sands BE (2018). ACG Clinical Guideline: Management of Crohn’s Disease in Adults. Am J Gastroenterol.

[3] Jabłońska B, Mrowiec S (2023). Nutritional Status and Its Detection in Patients with Inflammatory Bowel Diseases. Nutrients.

[4] Valvano M, Capannolo A, Cesaro N, Stefanelli G, Fabiani S, Frassino S (2023). Nutrition, Nutritional Status, Micronutrients Deficiency, and Disease Course of Inflammatory Bowel Disease. Nutrients.

[5] Gold SL, Rabinowitz LG, Manning L, Keefer L, Rivera-Carrero W, Stanley S (2023). High Prevalence of Malnutrition and Micronutrient Deficiencies in Patients with Inflammatory Bowel Disease Early in Disease Course. Inflamm Bowel Dis.

[6] Pulley J, Todd A, Flatley C, Begun J (2020). Malnutrition and quality of life among adult inflammatory bowel disease patients. JGH Open.

[7] Scaldaferri F, Pizzoferrato M, Lopetuso LR, Musca T, Ingravalle F, Sicignano LL (2017). Nutrition and IBD: Malnutrition and/or Sarcopenia? A Practical Guide. Gastroenterol Res Pract.

[8] Parra Izquierdo L V, Orduz-Diaz G, Frías-Ordoñez J, Hoyos-Rondon S, Reatiga-Pedraza A, Sarmiento-Navarro J (2024). P563 Nutritional screening in patients with inflammatory bowel disease: a challenge in the timely diagnosis of malnutrition and sarcopenia. J Crohn’s Colitis.

[9] Li S, Ney M, Eslamparast T, Vandermeer B, Ismond KP, Kroeker K (2019). Systematic review of nutrition screening and assessment in inflammatory bowel disease. World J Gastroenterol.

[10] Dua A, Corson M, Sauk JS, Jaffe N, Limketkai BN (2023). Impact of malnutrition and nutrition support in hospitalised patients with inflammatory bowel disease. Aliment Pharmacol Ther.

[11] Zhang J, Xu W, Zhang H, Fan Y (2024). Association between risk of malnutrition defined by patient-generated subjective global assessment and adverse outcomes in patients with cancer: a systematic review and meta-analysis. Public Health Nutr.

[12] Matsui R, Rifu K, Watanabe J, Inaki N, Fukunaga T (2023). Impact of malnutrition as defined by the GLIM criteria on treatment outcomes in patients with cancer: A systematic review and meta-analysis. Clin Nutr.

[13] Hashash JG, Elkins J, Lewis JD, Binion DG (2024). AGA Clinical Practice Update on Diet and Nutritional Therapies in Patients With Inflammatory Bowel Disease: Expert Review. Gastroenterology.

[14] Sandhu A, Mosli M, Yan B, Wu T, Gregor J, Chande N (2016). Self-Screening for Malnutrition Risk in Outpatient Inflammatory Bowel Disease Patients Using the Malnutrition Universal Screening Tool (MUST). J Parenter Enter Nutr.

[15] Jansen I, Prager M, Valentini L, Büning C (2016). Inflammation-driven malnutrition: A new screening tool predicts outcome in Crohn’s disease. Br J Nutr.

[16] Jimenez EY, Lamers-Johnson E, Long JM, Woodcock L, Bliss C, Steiber AL (2024). Predictive Validity of the Academy of Nutrition and Dietetics/American Society for Parental Nutrition Indicators to Diagnose Malnutrition and the Screening Tool for Risk on Nutritional Status and Growth among Hospitalized Children Relative to Medical Outcomes. J Pediatr.

[17] Irvine EJ (1999). Development and subsequent refinement of the inflammatory bowel disease questionnaire: A quality-of-life instrument for adult patients with inflammatory bowel disease. J Pediatr Gastroenterol Nutr.

[18] König HH, Ulshöfer A, Gregor M, Von Tirpitz C, Reinshagen M, Adler G (2002). Validation of the EuroQol questionnaire in patients with inflammatory bowel disease. Eur J Gastroenterol Hepatol.

[19] Castro-Vega I, Veses Martín S, Cantero Llorca J, Barrios Marta C, Bañuls C, Hernández-Mijares A (2018). Validity, efficacy and reliability of 3 nutritional screening tools regarding the nutritional assessment in different social and health areas. Med Clin.

[20] Ortíz-Gutiérrez S, Pérez-Cruz E, Lara-Pompa NE, Serralde-Zúñiga AE, Fewtrell M, Peralta-Pedrero ML (2019). Validation and Adaptation of the Spanish Version of the STRONGkids Nutrition Screening Tool. Nutr Clin Pract.

[21] Kwon SJ, Khan MS, Kim SG (2024). Intestinal Inflammation and Regeneration-Interdigitating Processes Controlled by Dietary Lipids in Inflammatory Bowel Disease. Int J Mol Sci.

[22] Massironi S, Viganò C, Palermo A, Pirola L, Mulinacci G, Allocca M (2023). Inflammation and malnutrition in inflammatory bowel disease. Lancet Gastroenterol Hepatol.

[23] Kaczmarczyk O, Dąbek-Drobny A, Piątek-Guziewicz A, Woźniakiewicz M, Paśko P, Dobrowolska-Iwanek J (2022). The Importance of Nutritional Aspects in the Assessment of Inflammation and Intestinal Barrier in Patients with Inflammatory Bowel Disease. Nutrients.

[24] Fiorindi C, Dragoni G, Scaringi S, Staderini F, Nannoni A, Ficari F (2021). Relationship between nutritional screening tools and glim in complicated IBD requiring surgery. Nutrients.

[25] Lin A, Micic D (2021). Nutrition Considerations in Inflammatory Bowel Disease. Nutr Clin Pract.

[26] Ünal NG, Oruç N, Tomey O, Özütemiz AÖ (2021). Malnutrition and sarcopenia are prevalent among inflammatory bowel disease patients with clinical remission. Eur J Gastroenterol Hepatol.

[27] Einav L, Hirsch A, Ron Y, Cohen NA, Lahav S, Kornblum J (2021). Risk factors for malnutrition among ibd patients. Nutrients.

[28] Taylor LM, Eslamparast T, Farhat K, Kroeker K, Halloran B, Shommu N (2021). Using Patient Completed Screening Tools to Predict Risk of Malnutrition in Patients with Inflammatory Bowel Disease. Crohn’s Colitis 360.

[29] Fuller MK (2019). Pediatric Inflammatory Bowel Disease: Special Considerations. Surg Clin North Am.

[30] Parra RS, Chebli JMF, Amarante HMBS, Flores C, Parente JML, Ramos O (2019). Quality of life, work productivity impairment and healthcare resources in inflammatory bowel diseases in Brazil. World J Gastroenterol.

[31] García-Gamboa R, Díaz-Torres O, Gradilla-Hernández MS, Pérez-Brocal V, Moya A, González-Avila M (2024). Gut Bacterial Composition and Nutritional Implications in Mexican and Spanish Individuals with Inflammatory Bowel Disease Compared to Healthy Controls. Int J Mol Sci.

[32] Nascimento R de P do, Moya AMTM, Machado AP da F, Geraldi MV, Diez-Echave P, Vezza T (2021). Review on the potential application of non-phenolic compounds from native Latin American food byproducts in inflammatory bowel diseases. Food Res Int.

[33] Velasco Rodríguez-Belvís M, Palomino L, Pujol-Muncunill G, Martin-Masot R, Medina Benítez E, Fernández-Lorenzo AE (2024). Transcultural adaptation and validation of IMPACT-III and IMPACT-III-P in Spanish families: a multicenter study from SEGHNP. Eur J Pediatr.

[34] Cox SR, Czuber-Dochan W, Wall CL, Clarke H, Drysdale C, Lomer MC (2022). Improving Food-Related Quality of Life in Inflammatory Bowel Disease through a Novel Web Resource: A Feasibility Randomised Controlled Trial. Nutrients.

